# Editorial: Addressing gender inequality in healthcare leadership: a path to enhanced patient outcomes

**DOI:** 10.3389/fpubh.2026.1834414

**Published:** 2026-04-14

**Authors:** Mustafa F. Özbilgin, Aline Yacoubian, Deborah Mukherji, Alice Dragomir, Albert El Hajj

**Affiliations:** 1 Brunel Business School, Brunel University of London, London, United Kingdom; 2 American University of Beirut Medical Center, Beirut, Lebanon; 3 Department of Hematology Oncology, Clemenceau Medical Center, Dubai, United Arab Emirates; 4 Faculté de Pharmacie, Université de Montréal, Montreal, QC, Canada

**Keywords:** gender, gender equality, health, healthcare, women

Women continue to be underrepresented and undervalued in top positions in research and medicine, where they encounter multiple obstacles and ongoing gender bias (Yacoubian et al.). Gender inequality in healthcare is often framed primarily in terms of representation. For example, the nursing profession is greatly influenced by gender norms and stereotypes, which sustain structural injustices that have a detrimental effect on both professional experiences and the effectiveness of the healthcare system. These employment difficulties are made worse in Türkiye by systemic injustices and patriarchal standards, especially for female nurses (Aca et al.).

Female physicians from Türkiye were more likely to report experiencing gender discrimination in the workplace, especially when it came to the burden of social roles, opportunities for career advancement, and leadership roles. They also reported that male physicians' discriminatory, negative, and discouraging attitudes had a major influence on their career choices (Ocakli et al.). Female physicians have been subject of higher sexual harassment than men in a survey from the American Society of Anesthesiologists in the United States with more burnout feelings and/or declining a job opportunity or intention of leaving the workplace (Shenoy et al.).

Women are also at higher risk of perception of health conditions such as perception of menstrual pain or menstrual-related symptoms and disorders, particularly dysmenorrhea (Sánchez-López et al.). They were at higher risk of fear during the COVID-10 pandemic, thus, requesting that reliable information be made available them as they are heavily impacted by the pandemic to lessen the anxiety brought on by this impression (Kang et al.). This was also noted in Lebanon wherein Lebanese women experienced higher rates of burnout compared to men during the pandemic (Abi-Gerges et al.).

Addressing gender inequality requires systemic institutional change. When it comes to incorporating gender issues into health policy formulation, policy actor leadership is crucial and hence implementing health policies in an equitable and efficient manner requires bolstering institutional processes and data systems for gender-responsive planning (Odero et al.). Reframing gender disparity as a feature of institutional architecture also highlights the broader societal implications of gender equality in healthcare leadership as health systems are central to addressing global challenges ranging from demographic change to public health crises. This entails human resources working toward fair, supportive and inclusive health workforce and education policies, professional and public communication initiatives to raise awareness of the advantages of gender equality and equal pay (Abi-Gerges et al.; Davies and Yarrow). Personalizing screening recommendations for certain morbidities such as breast cancer could enhance efficiency, support timely detection, and optimize resource use (Lim et al.).

Gender inequality in healthcare reflects the organizational structures through which authority, expertise, and care work are organized. To understand persistent disparities in healthcare leadership and professional recognition, it is therefore necessary to examine what we term the Gendered Institutional Architecture of Care (GIAC) as displayed in Figure 1.

**Figure 1:**
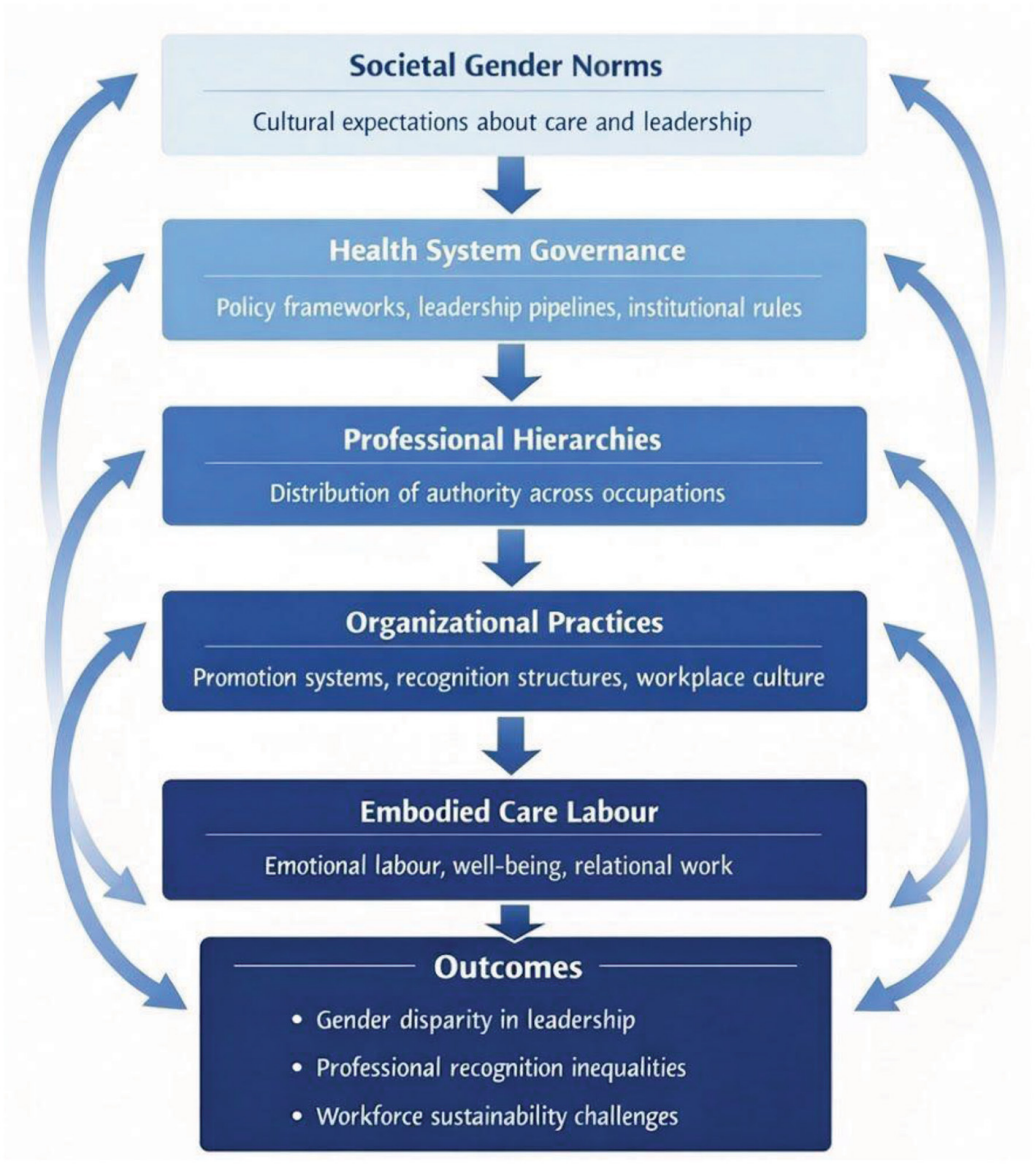
Gendered institutional architecture of care framework.

The concept of GIAC refers to the layered institutional arrangements through which power, professional legitimacy, and care labor are structured within healthcare systems. This architecture operates across multiple interacting levels, including societal gender norms, health system governance, professional hierarchies, organizational practices, and embodied experiences of healthcare work. Together these levels shape how authority is distributed, how leadership is recognized, and how care labor is valued within healthcare institutions.

Organizational rules and evaluation systems often reflect historically masculine models of professional commitment that privilege uninterrupted career trajectories, continuous availability, and long working hours. These norms shape perceptions of leadership legitimacy and influence whose careers align with dominant expectations of professional success. Within healthcare organizations, leadership authority has historically been concentrated in occupational pathways associated with medical authority and institutional governance.

Although women constitute a large proportion of the global healthcare workforce, their representation decreases markedly in positions associated with strategic decision-making and institutional power. In healthcare systems, access to leadership often depends on forms of capital associated with particular professional trajectories, institutional networks, and recognition structures. When these forms of capital are unevenly distributed across gender lines, professional hierarchies reproduce gendered patterns of authority.

Proposition 1: Gender disparities in healthcare leadership emerge from the interaction between professional hierarchies and gendered institutional norms embedded within healthcare governance structures.

Healthcare work is also shaped by embodied experiences that influence wellbeing, professional identity, and organizational recognition. Healthcare labor is physically demanding, emotionally intensive, and frequently performed under conditions of organizational pressure and resource constraints. When institutional governance systems fail to recognize the embodied dimensions of care work, they reinforce forms of invisibility that disproportionately affect those performing such labor.

Proposition 2: The institutional undervaluation of care labor contributes to gender inequality by limiting recognition, authority, and career progression for roles associated with relational and emotional work.

Viewing gender inequality through the lens of the GIAC highlights the structural nature of disparities within healthcare systems. Gender inequality is not simply the result of individual career trajectories but emerges from interacting institutional layers that shape opportunities, recognition, and authority. These layers include societal gender norms, governance structures, professional hierarchies, organizational practices, and the embodied realities of healthcare work.

## Future research agenda

Comparative studies across health systems can examine how different governance models shape gendered institutional architectures. Research on professional capital and leadership pathways can deepen understanding of how symbolic and institutional capital operate within healthcare fields. Studies of embodied labor and organizational wellbeing can explore how recognition of care labor influences workforce sustainability and professional identity. Interdisciplinary scholarship linking organizational theory, public health, and feminist political economy can further develop conceptual tools for analyzing gender inequality within complex healthcare systems.

